# Zedoary oil (*Ezhu You*) inhibits proliferation of AGS cells

**DOI:** 10.1186/1749-8546-8-13

**Published:** 2013-06-28

**Authors:** Hailian Shi, Bao Tan, Guang Ji, Lan Lu, Aili Cao, Songshan Shi, Jianqun Xie

**Affiliations:** 1 Institute of Digestive Disease, Longhua Hospital, Shanghai University of Traditional Chinese Medicine, 725 South Wanping Road, XuHui District, Shanghai 200032, PR China; 2 Institute of Materia Medica, Shanghai University of Traditional Chinese Medicine, 1200 Cailun Road, Zhangjiang Hi-tech Park, Shanghai 201203, PR China; 3 Shanghai Key Laboratory of Complex Prescription, 1200 Cailun Road, Zhangjiang Hi-tech Park, Shanghai 201203, PR China; 4 Chinese Medicine Hospital of Shanxi Province, Taiyuan, Shanxi Province 030012, PR China

## Abstract

**Background:**

Zedoary (*Curcumae Rhizoma*, *Ezhu*), a Chinese medicinal herb, has been reported to show anticancer activity. This study aims to investigate the effect of zedoary oil (*Ezhu You*) on the proliferation of AGS cells which is one gastric cancer cell line.

**Methods:**

The main ingredients of the herb were detected by GC-MS for herbal quality control. Cell viability was measured by MTT assay and cell proliferation was investigated by immunocytochemical staining for proliferating cell nuclear antigen (PCNA) protein. In addition, the cell cycle distributions were detected by flow cytometry with propidium iodine (PI) staining and the apoptosis rates were evaluated by flow cytometry with annexin V/PI double-staining. The morphological changes associated with apoptosis were observed by Hoechst 33342/PI double-staining. Protein expression was determined by western blot analysis.

**Results:**

The main ingredients of the herb, including curzerene (26.45%), eucalyptol (12.04%), curcumol (9.04%), pyridine (7.97%), germacrone (7.89%), β-elemene (7.36%), τ-elemene (4.11%) and 28 other ingredients, including curdione, were consistent with the chemical profiles of zedoary. Zedoary oil significantly decreased the cell viability of AGS cells (*P* < 0.01) and MGC 803 cells (*P* < 0.01), and the inhibitory effects were attenuated by elevated concentrations of FBS. At high concentrations (≥90 μg/mL), zedoary oil killed GES-1 cells. At low concentrations (≤60 μg/mL), zedoary oil was less inhibitory toward normal gastric epithelial cells than gastric cancer cell lines. In AGS cells, zedoary oil inhibited cell proliferation in a dose- and time-dependent manner, with decreased PCNA protein expression in the zedoary oil-treated cells, and arrested the cell cycle at S, G_2_/M and G_0_/G_1_ stages after treatment for 6–48 h. At concentrations of 30, 60 and 90 μg/mL, which resulted in significant inhibition of proliferation and cell cycle arrest, zedoary oil induced cell apoptosis. In addition, Hoechst 33342/PI double-staining confirmed the morphological characteristics of cell apoptosis at 24 h. Zedoary oil upregulated the ratio of Bax/Bcl-2 protein expression (*P* < 0.01).

**Conclusions:**

Zedoary oil inhibited AGS cell proliferation through cell cycle arrest and cell apoptosis promotion, which were related to Bax/Bcl-2 protein expression.

## Background

*Curcuma phaeocaulis*, *Curcuma kwangsiensis* and *Curcuma wenyujin* are named zedoary in the *Chinese Pharmacopoeia* and used as antiviral and antimicrobial medicines [1-3]. Zedoary oil is a Chinese medicine that is used for treatment of gynecologic inflammation [4], pneumonia [5], pediatric diseases [6], viral myocarditis [7] and malignant tumors, such as oophoroma, hepatocellular carcinoma and lung cancer [8-11]. Moreover, zedoary oil is a safe drug with low toxicity [12]. Deng *et al.*[13] reported that the acute toxicity of zedoary turmeric oil gelatin microspheres might result from dystopic embolism rather than the zedoary turmeric oil itself entrapped in the microspheres.

Zedoary belongs to the *Zingiberacea* family, which is composed of about 70 species of rhizomatous herbs at home and abroad, with approximately 20 species existing in China [1,10]. Different species of zedoary and different preparations have different chemical ingredients [14], which result in different biological actions. Many chemical analysis methods, including thin-layer chromatography scanning, high-performance liquid chromatography, gas chromatography (GC) and gas chromatography–mass spectrometry (GC-MS), are used to detect the chemical compounds in essential oil of zedoary and also for quality control [15].

Gastric glandular cells are replaced by intestinal-type epithelial cells with high cell proliferation rates and fibrosis in severe inflammation of the gastric membrane, resulting in chronic atrophic gastritis with intestinal metaplasia and dysplasia, imbalance between cell proliferation and apoptosis in the normal gastric mucosa and increasing incidence of gastric cancer [16-19].

Proliferating cell nuclear antigen (PCNA) was originally identified as an antigen expressed in the nuclei of cells during the DNA synthesis phase of the cell cycle [20], and only exists in normal proliferative cells and cancer cells.

Bax and Bcl-2 are very important for cytochrome c-dependent apoptosis. Bax inserts itself into the outer mitochondrial membrane, followed by cytochrome c release from mitochondria. In contrast, when Bcl-2 binds to the outer mitochondrial membrane, the release of cytochrome c is blocked [21,22]. Many anticancer agents can induce release of cytochrome c by upregulating Bax expression and/or downregulating Bcl-2 expression [23-25].

Chinese medicines are available for treatment of patients with chronic atrophic gastritis [26]. Zedoary-containing Chinese herbal formulas, *e.g.*, *Weiqi* decoction (an empirical formula from Longhua Hospital, Shanghai University of Traditional Chinese Medicine, China), are often used for treatment of gastric diseases [27-29]. However, the effects of zedoary oil on gastric epithelial cells with high proliferation rates are unclear.

The AGS cell line, a type of human gastric cancer epithelial cell line, is used as a cell model for abnormal proliferation and apoptosis in the gastric mucosa and gastric cancer research [30,31]. The present study aims to investigate the effect of zedoary oil (*Ezhu You*) on AGS cell proliferation.

## Methods

### Materials

Zedoary oil was purchased from Shanghai Institute for the National Institute for the Control of Pharmaceutical and Biological Products (Lot No. 111544-200703) (Shanghai, China). AGS (TCHu 7) and MGC 803 (TCHu 84) cell lines were purchased from Cell Bank of Academia Sinica (Shanghai, China). The GES-1 cell line was purchased from Cell Bank of Chinese Academy of Medical Sciences (Beijing, China). Ham’s/F-12, DMEM/High Glucose and RPMI 1640 media were purchased from Thermo Fisher Scientific Inc. (IL, USA). Fatal bovine serun (FBS) was purchased from Hangzhou Sijiqing Biological Engineering Materials Co. Ltd. (Hangzhou, China). FBS (Lot No. 989268) was purchased from Gibco (NY, USA). DMSO (Lot No. 1988B176) was purchased from Amresco (OH, USA). MTT (Lot No. 091205) was purchased from Richu BioScience Co. Ltd. (Shanghai, China). PI (Lot No. 118 K3538) and DMSO (Lot No. 019 K2300) were purchased from Sigma Chemical Co. (MO, USA). A Hoechst 33342/PI Apoptosis/death Staining Kit (Catalogue No. C1056) and Hematoxylin Staining Kit (Catalogue No. C0107) were purchased from Beyotime (Shanghai, China). Trypsin (Lot No. 632461) was purchased from Invitrogen (CA, USA). RNase A (Lot No. 3408B040) was purchased from Beijing Jingkehongda Biotech. Co. Ltd. (Beijing, China). Annexin V (Lot No. 40601) was purchased from BioVision Inc. (CA, USA). Anti-Bax (Lot No. 4), anti-Bcl-2 (Lot No. 2) and anti-β-actin (Lot No. 3) antibodies were purchased from Cell Signaling Technology (MA, USA). An anti-PCNA (Lot No. YJ020304CS) antibody was purchased from Epitomics (CA, USA). An S_ABC_ Kit (Lot No. 06L06AJ) and a DAB Kit (Catalogue No. AR1022) were purchased from Wuhan Boster Biological Technology Co. Ltd. (Wuhan, China). An ECL plus Kit (Lot No. 84A) was purchased from GE Healthcare (NA, UK). Cell plates were purchased from Greiner Bio-One (Frickenhausen, Germany). Syringe filters were purchased from Pall Co. Ltd. (MI, USA).

### Identification of the main constituents in zedoary oil by GC-MS analysis

Zedoary oil was diluted with chloroform at a ratio of 1:1000. The analysis was performed on a Thermo Scientific system composed of a DSQ mass spectrometer coupled with a Trace GC Ultra gas chromatograph and an AS 3000 autosampler (Thermo Scientific, USA). The GC was equipped with a 30-m (0.25-mm internal diameter; 0.25-μm film thickness) TR-5MS fused-silica capillary column (Thermo Scientific). The splitless injection port temperature was set at 250°C. The column temperature program was 50°C for 1 min, followed by elevation to 110°C at 5°C/min, 140°C at 3°C/min and 170°C at 5°C/min. Finally, the temperature was raised to 230°C at 3°C/min and held at 230°C for 5 min. The constant flow rate was 1 mL/min He. The MS was operated in the positive EI mode. The ion source temperature was set at 250°C. The peaks were identified by comparisons with The National Institute of Standards and Technology (NIST) library. Relative quantitative data were obtained from the normalized peak areas: % area = (area/total area) × 100.

### Cell culture

AGS cells, MGC 803 cells (gastric cancer cell line) and GES-1 cells (normal gastric epithelial cells) were cultured in Ham’s/F-12, RPMI 1640 and DMEM media supplemented with 10% FBS, respectively, at 37°C in a 5% CO_2_ atmosphere.

### Proliferation assay

Cells were seeded in 96-well culture plates at a density of 1.0 × 10^4^ cells/mL in 200 μL of medium and allowed to adhere to the plates overnight. The cells were then treated with a range of concentrations (0–300 μg/mL) of zedoary oil or 0.1% DMSO for 24, 48 or 72 h. Subsequently, 20 μL of MTT was added to each well and incubated at 37°C for 4 h. After removal of the medium and MTT, 150 μL of DMSO was added to each well and shaken for 15 min to completely dissolve the formazan crystals. Finally, the absorbances at 570 nm of the dissolved solutions were detected using a SpectraMAX 190 microplate reader (Molecular Devices, USA). The cell viability rate was calculated using the following equation: rate %) = (treated absorbance/untreated absorbance) × 100. The effects of three compounds in zedoary oil, namely beta-elemene, curcumol and curdione, on the proliferation of AGS cells were also investigated.

### Immunocytochemical staining for PCNA protein

After treatment with zedoary oil for 24 h, AGS cells were washed with 1× PBS, and fixed in 4% paraformaldehyde for 20 min. The cell membranes were permeated with 0.5% Triton X-100 (pH 7.4) for 20 min at room temperature followed by incubation with 3% H_2_O_2_ for 15 min. After blocking with 5% BSA for 20 min at 37°C, the cells were incubated with a rabbit anti-PCNA monoclonal antibody and visualized using the S_ABC_ Kit and DAB Kit. The cell nuclei were stained with hematoxylin. Finally, coverslips were placed on the glass slides on a drop of mounting medium, and the cells were photographed under a microscope (Olympus CKX41, Japan).

### Cell cycle distribution analysis

Cells were plated in 6-well culture plates at a density of 2.0 × 10^4^ cells/mL in 3 mL of medium and allowed to adhere to the plates overnight. Subsequently, the medium containing 10% FBS was removed and the same volume of medium without FBS was added for 24 h. The cells were then incubated with a range of concentrations (30, 60 and 90 μg/mL) of zedoary oil or 0.1% DMSO in medium containing 10% FBS for 6, 12, 24 or 48 h. After the treatments, the cells were harvested by trypsinization, washed twice with PBS, fixed overnight with 70% cold ethanol and stained with PI solution containing 50 μg/mL RNase A and 0.1% Triton X-100. The cell cycle distributions were detected using a FACScan flow cytometer (Becton Dickinson, USA), and the data were analyzed by ModFitLT V3.0 software (Becton Dickinson).

### Cell apoptosis/necrosis detection by annexin V/PI double-staining

Cells were plated in 6-well culture plates at a density of 2.5 × 10^4^ cells/mL in 3 mL of medium and allowed to adhere to the plates overnight. Next, the medium containing 10% FBS was removed and the same volume of medium without FBS was added for 24 h. The cells were then incubated with a range of concentrations (60, 90 and 120 μg/mL) of zedoary oil in medium containing 10% FBS for 24 h. After the treatments, the cells were harvested by careful trypsinization, washed twice with 1× annexin V binding buffer, resuspended in binding buffer and stained with annexin V and PI. Cell apoptosis was detected using the FACScan flow cytometer.

### Apoptosis and necrosis detection by Hoechst 33342/PI double-staining

Cells were seeded in 6-well culture plates at a density of 2.0 × 10^4^ cells/mL in 3 mL of medium and allowed to adhere to the plates overnight. The medium containing 10% FBS was removed and the same volume of medium without FBS was added for 24 h. The cells were then incubated with a range of concentrations (30, 60 and 90 μg/mL) of zedoary oil or 0.1% DMSO in medium containing 10% FBS for 24 h. After the treatments, the cells were harvested by careful trypsinization and resuspended in staining buffer. The cells were stained with Hoechst 33342 and PI, and analyzed for apoptosis and/or necrosis under a fluorescence microscope (Olympus CKX41, Japan). The rates of Hoechst 33342-positive cells, whose blue color was brilliant and aggregative, were analyzed by Image-Pro Plus 6.0 software (Media Cybernetics, USA).

### Western blot analysis

Cells were plated in 6-well culture plates at a density of 4.0 × 10^4^ cells/mL in 3 mL of medium and allowed to adhere to the plates overnight. The medium containing 10% FBS was removed and the same volume of medium without FBS was added for 24 h. The cells were then incubated with a range of concentrations (60, 90 and 120 μg/mL) of zedoary oil or 0.1% DMSO in medium containing 10% FBS for 24 h. After the incubations, the cells were collected, lysed with cell lysis buffer and sonicated three times for 15 s each. The cell lysates were centrifuged for 15 min at 14,000 × *g* and 4°C, and the supernatants were collected. The protein samples were separated by SDS-PAGE (15% separating gel and 5% stacking gel) and transferred onto Hybond-NC membranes by wet transfer. Subsequently, the NC membranes were blocked with 5% non-fat milk solution and incubated with the primary antibodies against Bax, Bcl-2 and β-actin overnight at 4°C. After washing with 1× TBST, the NC membranes were incubated with goat anti-rabbit IgG (HuaAn, China). The protein bands were visualized with the ECL plus Kit, scanned and analyzed with SmartView software (Furi, China).

### Statistical analysis

The data were represented by the mean ± standard deviation (SD). Significant differences among three or more data sets were analyzed by one-way ANOVA with Dunnett’s test using PrismDemo 4 software (GraphPad Software Inc., USA). Differences between two groups were analyzed by Student’s *t*-test. The PrismDemo software did not provide exact *P* values for ANOVA, and thus no exact *P* values were reported. Values of *P* < 0.05 were considered to indicate statistical significance.

## Results

### GC-MS analysis of zedoary oil for quality control

**Figure 1:**
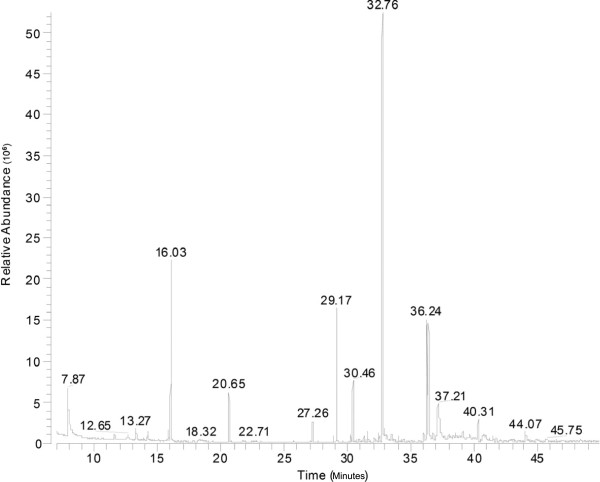
GC-MS spectrum of zedoary oil.

As shown in Figure 1 and Table 1, the GC-MS analysis detected 35 chemical compounds in zedoary oil. Curzerene (26.45%), eucalyptol (12.04%), curcumol (9.04%), pyridine (7.97%), germacrone (7.89%), β-elemene (7.36%), τ-elemene (4.11%), curdione (1.23%), δ-elemene (1.05%), (-)-alcanfor (3.18%), camphene (1.18%), 2-β-pinene (0.95%), bornylene (0.73%), β-cubebene (0.66%), α-caryophyllene (0.6%) and α-pinene (0.67%) were indicated as the main compounds of essential oil of zedoary, whose relative contents (% areas) were >0.5% in the total zedoary oil. Many other compounds were also detected by the GC-MS, and several chemical compounds could not be determined from the NIST library.

**Table 1:** Compounds detected in the zedoary oil

**Apex RT**	**Area (%)**	**Composition**	**AS No.**
7.87	7.97	pyridine	110-86-1
11.58	0.45	1,1-ethanediol diacetate	542-10-9
12.65	0.67	α-pinene	80-56-8
13.27	1.18	camphene	79-92-5
13.91	0.21	sabinene	3387-41-5
14.19	0.95	β-pinene	127-91-3
15.82	0.73	bornylene	464-17-5
16.03	12.04	eucalyptol	470-82-6
20.65	3.18	(-)-alcanfor	464-48-2
27.26	1.05	δ-elemene	20307-84-0
29.17	7.36	β-elemene	515-13-9
30.29	0.66	β-cubebene	13744-15-5
30.46	4.11	τ-elemene	30824-67-0
30.72	0.13	β-cubebene	13744-15-5
31.3	0.27	γ-muurolene	30021-74-0
31.44	0.16	γ-cadinene	39029-41-9
31.63	0.6	α-caryophyllene	6753-98-6
32.09	0.31		
32.24	0.07		
32.43	0.42	germacrene-D	23986-74-5
32.51	0.26	zingiberene	495-60-3
32.76	26.45	curzerene	17910-09-7
32.96	0.39	α-selinene	473-13-2
33.45	0.35	β-cadinene	523-47-7
34.02	0.34	(+)-β-guaiene	88-84-6
36.24	7.89	germacrone	6902-91-6
36.39	7.63		
36.78	0.36		
37.21	9.04	curcumol	4871-97-0
39.1	0.41	butylidenephtalide	551-08-6
40.31	1.23	curdione	13657-68-6
41.24	0.11		
41.53	0.24		
41.74	0.23		
44.07	1.13	2-[(4-methoxyphenyl)methylene]-Cyclohexanone	5765-29-7

### Inhibitory effects of zedoary oil on cell viability

Zedoary oil inhibited the proliferation of AGS cells in a dose- and time-dependent manner after treatment for 24, 48 and 72 h (*P* < 0.01 *vs.* control cells) (Table 2). The IC_50_ values of zedoary oil at 24, 48 and 72 h were 72.40, 64.28 and 63.83 μg/mL, respectively.

**Table 2:** Effects of zedoary oil and β-elemene on cell viability

**Group**	**n**	**Cell viability**		
		**24 h**	**48 h**	**72 h**
Zedoary oil				
0 μg/mL	4	99.5 ± 0.9	99.8 ± 0.3	99.9 ± 0.2
1 μg/mL	4	95.8 ± 2.9	82.5 ± 2.5**	71.0 ± 7.0**
10 μg/mL	4	95.0 ± 2.3	77.1 ± 6.2**	69.2 ± 2.3**
30 μg/mL	4	81.2 ± 6.2**	74.3 ± 3.5**	58.4 ± 2.9**
60 μg/mL	4	65.3 ± 5.1**	49.5 ± 4.2**	42.5 ± 5.4**
90 μg/mL	4	47.4 ± 1.5**	19.0 ± 3.6**	16.7 ± 5.9**
100 μg/mL	4	35.4 ± 4.7**	13.7 ± 9.4**	9.8 ± 5.7**
300 μg/mL	4	14.8 ± 2.5**	5.3 ± 0.6**	2.5 ± 0.7**
β-elemene				
11.5 μg/mL	3	98.2 ± 2.0	98.1 ± 10.7	91.2 ± 7.4
23 μg/mL	4	97.1 ± 5.0	89.0 ± 8.5	86.8 ± 14.4
92 μg/mL	4	94.1 ± 6.8	81.6 ± 3.1*	79.6 ± 3.5*
184 μg/mL	4	87.6 ± 2.0	60.3 ± 9.5**	70.0 ± 9.1**
368 μg/mL	4	25.1 ± 2.4**	14.0 ± 6.3**	12.0 ± 3.4**
1104 μg/mL	4	9.3 ± 1.3**	3.2 ± 0.7**	5.0 ± 4.1**

AGS cells were treated with zedoary oil in Ham’s/F-12 medium containing 10% FBS for 24, 48 and 72 h. The cell viability was detected by MTT assays. *P < 0.05, **P < 0.01 *vs.* control cells.

Beta-elemene had inhibitory effects on AGS cell proliferation (*P* < 0.05, *P* < 0.01 for different concentration *vs.* control cells), and its IC_50_ values at 24, 48 and 72 h were 280.57, 212.98 and 243.98 μg/mL, respectively. Curcumol and curdione did not show significant inhibitory effects on AGS cell proliferation.

**Figure 2:**
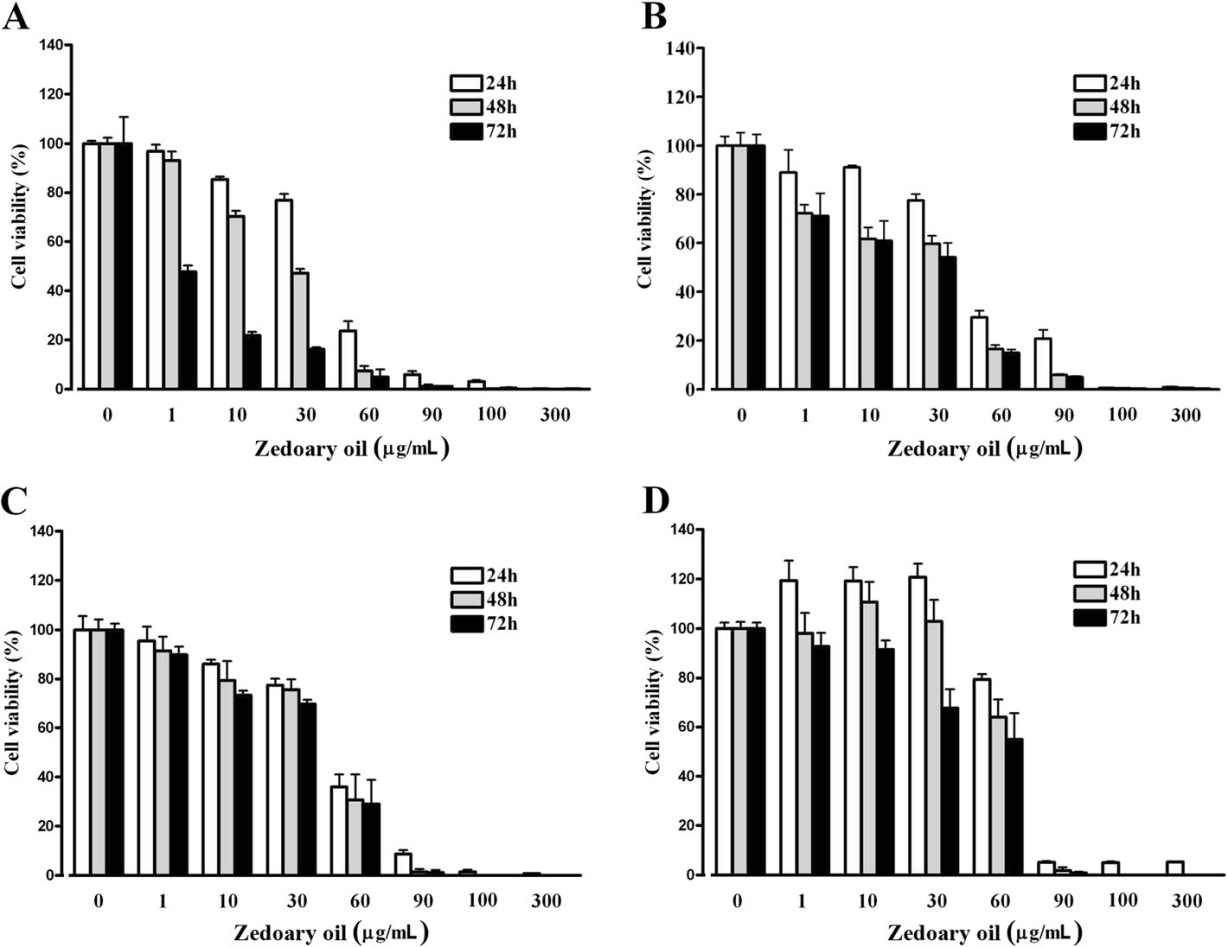
**Effects of zedoary oil on the viability of gastric cancer cells and normal gastric epithelial cells. (A)** AGS cells were treated with zedoary oil without FBS for 24, 48 and 72 h. **(B)** AGS cells were treated with zedoary oil containing 3% FBS for 24, 48 and 72 h. **(C)** MGC 803 cells were treated with zedoary oil containing 3% FBS for 24, 48 and 72 h. **(D)** GES-1 cells were treated with zedoary oil containing 3% FBS for 24, 48 and 72 h. Data represent means ± SD (N = 3).

As shown in Figure 2, zedoary oil had significant inhibitory effects on the proliferation of MGC 803 cells. Zedoary oil increased the proliferation of GES-1 cells at 1, 10 and 30 μg/mL from 24 to 48 h. After 72 h of treatment, zedoary oil showed low inhibitory effects on cell proliferation. At the concentration of 60 μg/mL, zedoary oil had low inhibitory effects on the viability of GES-1 cells. However, zedoary oil killed most of the AGS, MGC 803 and GES-1 cells at 90 μg/mL.

In the presence of different FBS concentrations (0, 3 and 10%), zedoary oil achieved different inhibitory effects on the proliferation of AGS cells. There was a negative correlation between the FBS concentration and the inhibitory effect (Figure 2).

### Inhibitory effects of zedoary oil on PCNA protein expression

**Figure 3:**
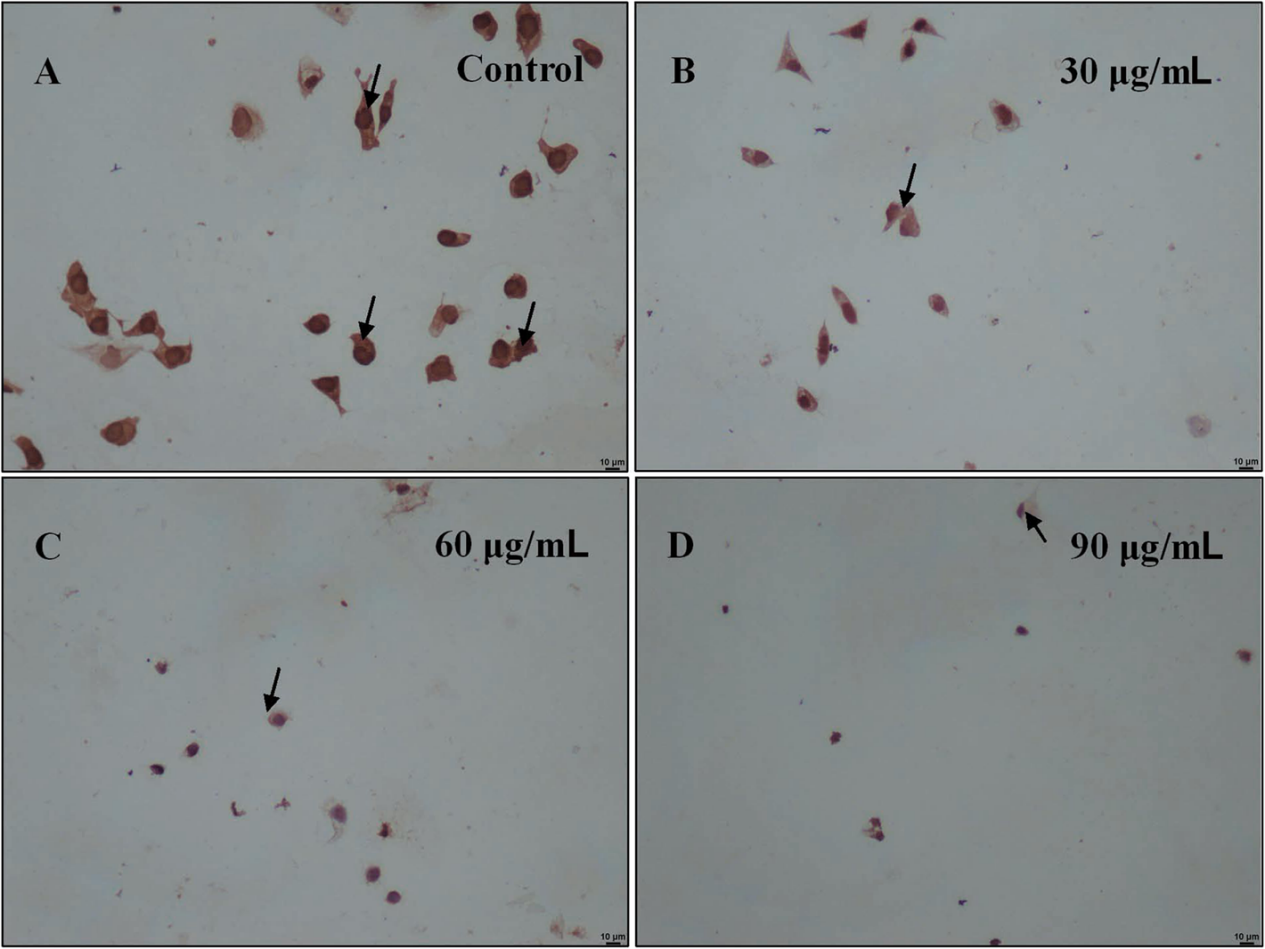
**Zedoary oil downregulates PCNA protein expression in AGS cells. (A-D)** Cells were treated with zedoary oil (0, 30, 60 and 90 μg/mL) for 24 h, respectively. The brown color represents positive expression of PCNA protein. The blue color represents the cell nuclei of AGS cells (200×). The control cells were AGS cells cultured in Ham’s/F-12 medium containing 10% FBS and 0.1% DMSO.

As shown in Figure 3, zedoary oil significantly decreased PCNA protein expression in AGS cells.

### Zedoary oil induces cell cycle arrest

**Figure 4:**
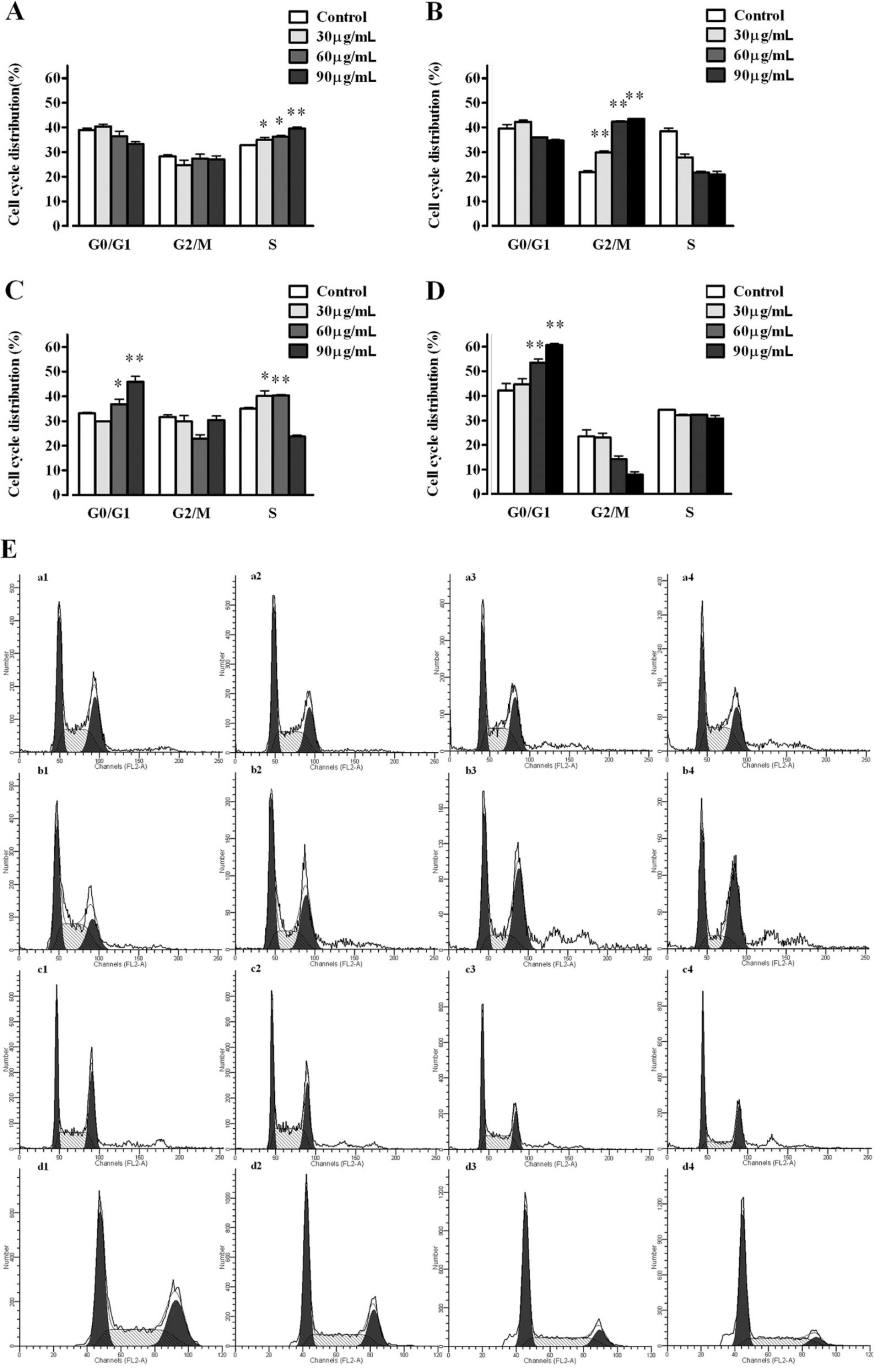
**Zedoary oil induces cycle arrest in AGS cells.** Zedoary oil was added at the indicated concentrations and the cells were incubated for 6, 12, 24 and 48 h. The cell cycle proportions were determined by flow cytometry after staining with PI. **(A–D)** Statistical analyses of the cell cycle phase distributions after 6, 12, 24 and 48 h, respectively. **(E)** Typical pictures of the respective cell cycle phase distributions from flow cytometry: a1–a4, control, 30, 60 and 90 μg/mL for 6 h; b1–b4, control, 30, 60 and 90 μg/mL for 12 h; c1–c4, control, 30, 60 and 90 μg/mL for 24 h; d1–d4, control, 30, 60 and 90 μg/mL for 48 h. The data represent means ± SD (*P < 0.05, **P < 0.01 *vs.* control cells). The control cells were AGS cells cultured in Ham’s/F-12 medium containing 10% FBS and 0.1% DMSO.

After treatment with zedoary oil at 60 and 90 μg/mL for 6 h, the population of cells in S phase increased from 32.74 (in control cells) to 39.59% (in 90 μg/mL-treated cells) (*P* < 0.05, *P* < 0.01 *vs.* control cells, respectively) (Figure 4A). After treatment for 12 h, the populations of cells in G_2_/M phase reached 29.86 (in 30 μg/mL-treated cells), 42.31 (in 60 μg/mL-treated cells) and 43.41% (in 90 μg/mL-treated cells), respectively, compared with 21.94% in the control group (*P* < 0.01, *P* < 0.01, *P* < 0.01 *vs.* control cells) (Figure 4B). After treatment for 24 h, the population of cells in G_0_/G_1_ phase was elevated from 33.20 (in control cells) to 45.88% (in 90 μg/mL-treated cells) (*P* > 0.05, *P* < 0.05, *P* < 0.01 *vs.* control cells) (Figure 4C), and the population of cells in S phase increased from 35.08 (in control cells) to 40.19 (in 30 μg/mL-treated cells) and 40.40% (in 60 μg/mL-treated cells) in zedoary oil-treated cells (*P* < 0.05, *P* < 0.01 *vs.* control cells), respectively. Zedoary oil (30, 60 and 90 μg/mL) elevated the population of cells in G_0_/G_1_ phase from 42.25 (in control cells) to 60.64% (in 90 μg/mL-treated cells) after treatment for 48 h (*P* > 0.05, *P* < 0.01, *P* < 0.01 *vs.* control cells, respectively) (Figure 4D).

### Zedoary oil induces cell apoptosis/necrosis

**Figure 5:**
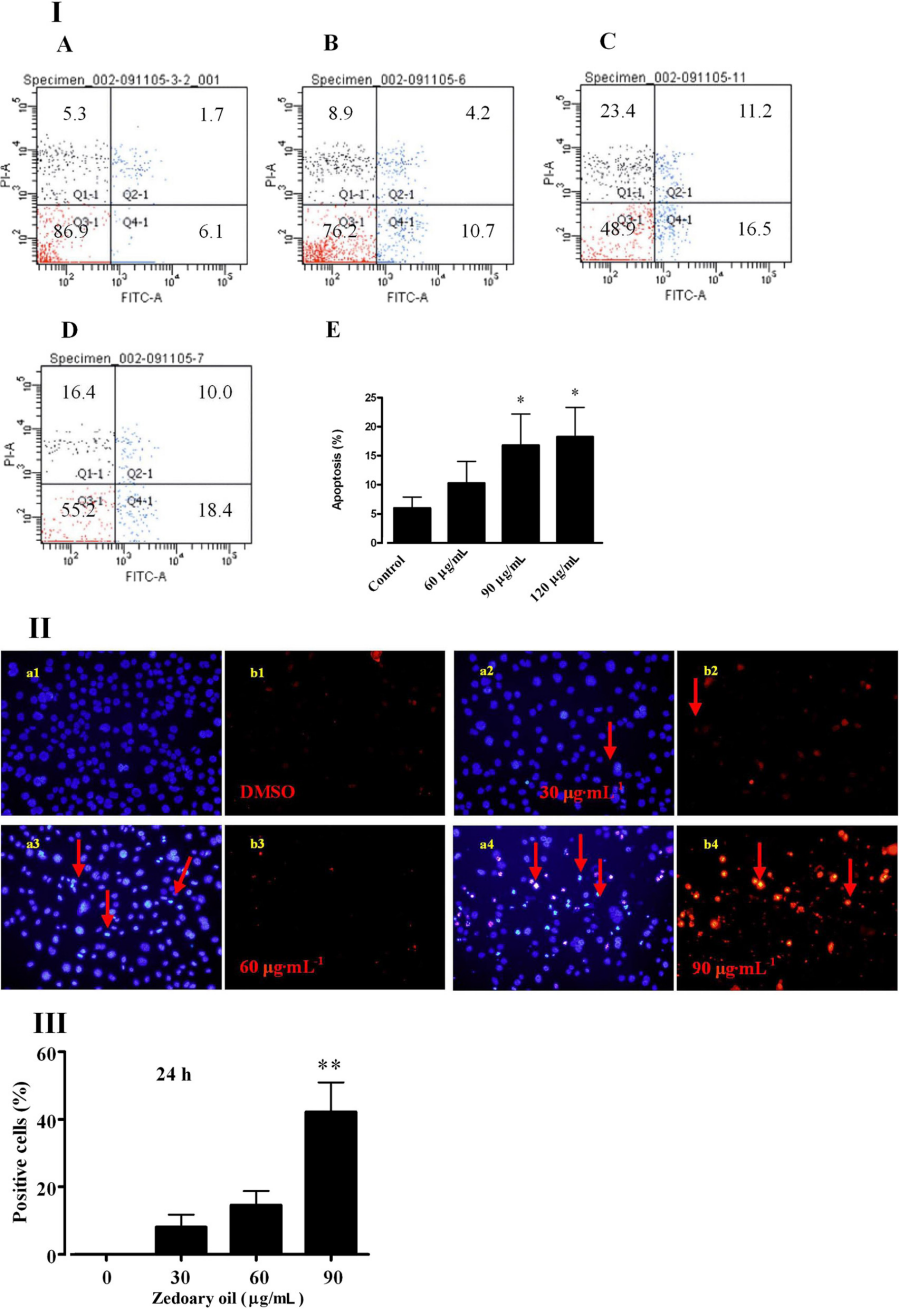
**Effects of essential oil of zedoary on cell apoptosis of AGS cells at 24 h. (IA)** Control; **(IB)** 60 μg/mL; **(IC)** 90 μg/mL; **(ID)** 120 μg/mL; **(IE)** effects of essential oil of zedoary on cell apoptosis evaluated by annexin V/PI double-staining at 24 h. **(II)** Effects of zedoary oil on AGS cell apoptosis and necrosis evaluated by Hoechst 33342/PI double-staining for 24 h (200×). **(III)** Ratios of Hoechst 33342-positive cells whose blue color staining was brilliant and aggregative. The control cells were AGS cells cultured in Ham’s/F-12 medium containing 10% FBS and 0.1% DMSO. *P < 0.05, **P < 0.01 *vs.* control cells.

Zedoary oil (60, 90 and 120 μg/mL) promoted the early cell apoptosis rate from 5.97 (in control cells) to 18.23% (in 120 μg/mL-treated cells), as detected by flow cytometry with annexin V/PI double-staining (*P* < 0.05 *vs.* control cells) (Figure 5I). The blue staining of Hoechst 33342, which crosses the cell membranes of both living and dying cells and stains their DNA, was of low intensity in untreated cells. However, the blue staining was brilliant and aggregative in treated cells, indicating that the DNA had become aggregative and that cell apoptosis was initiated after zedoary oil treatment. PI cannot penetrate the cell membranes of living cells, but can cross the cell membranes of dying cells and stain the DNA in the cell nucleus. Consequently, only late apoptotic and necrotic cells can be stained by PI. Thus, to distinguish late apoptotic and/or necrotic cells from early apoptotic cells, the numbers of Hoechst 33342-positive, but not PI-positive, cells were measured in this study. As shown in Figure 5, both cell apoptosis characteristics of brilliant and aggregative blue color staining were observed by Hoechst 33342/PI double-staining in zedoary oil-treated cells (90 μg/mL) for 24 h (*P* < 0.01 *vs.* control cells).

### Protein expressions of Bcl-2 and Bax

**Figure 6:**
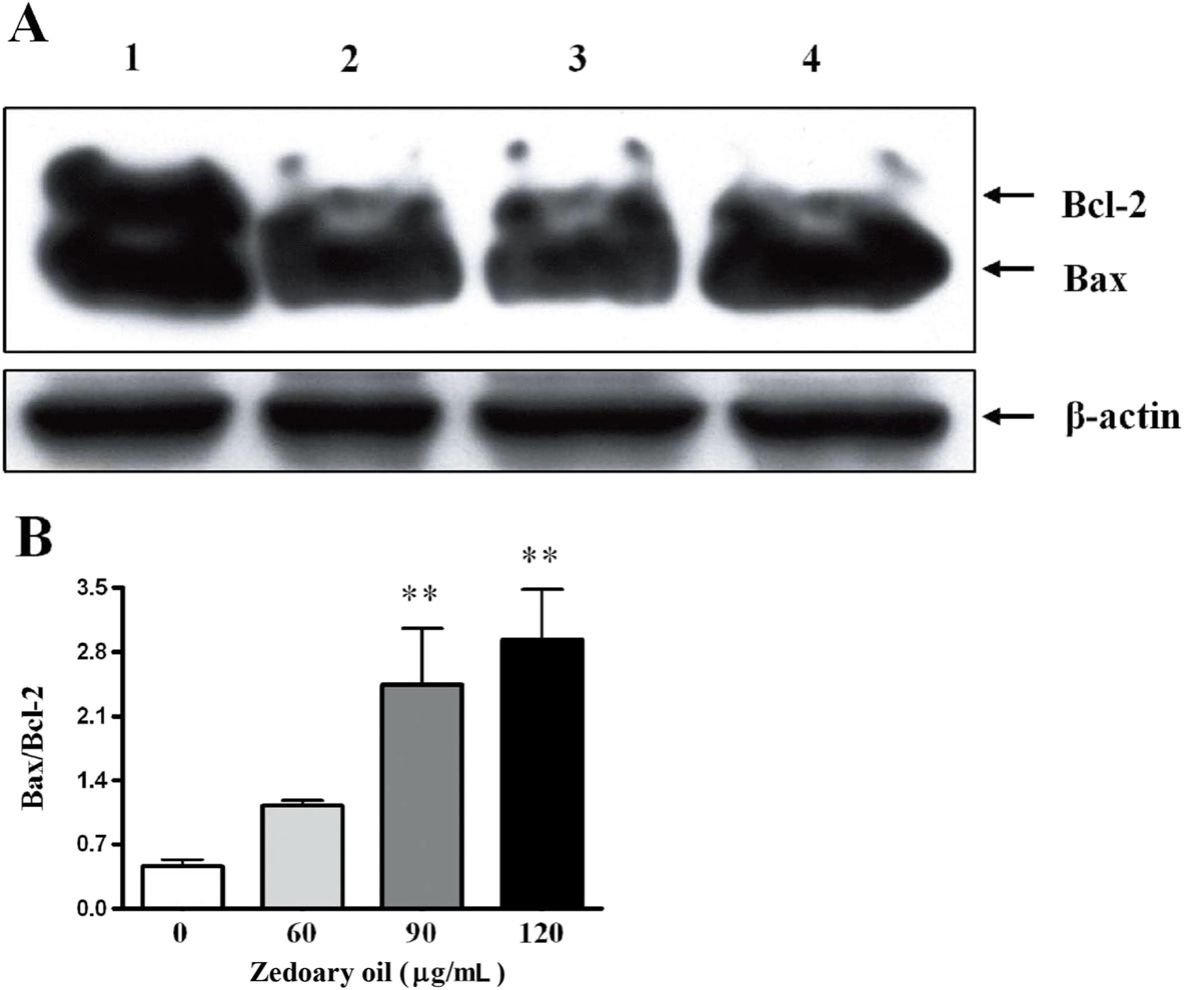
**Effects of zedoary oil on apoptosis-related Bcl-2 and Bax protein expression in AGS cells. (A)** Effects of zedoary oil on apoptosis-related protein Bcl-2 and Bax expression in AGS cells. The control cells were AGS cells cultured in Ham’s/F-12 medium containing 10% FBS and 0.1% DMSO. The cells were incubated with zedoary oil for 24 h, collected and measured for their Bax and Bcl-2 protein expression by western blot analysis. 1: control; 2: 60 μg/mL; 3: 90 μg/mL; 4: 120 μg/mL. **(B)** Effects of zedoary oil on the ratio of Bax/Bcl-2 expression.

The western blot analyses revealed that the Bcl-2 protein levels in AGS cells were significantly decreased by zedoary oil at 60, 90 and 120 μg/mL. Zedoary oil did not upregulate the Bax protein expression level at 60 and 90 μg/mL, but did increase the Bax protein expression level at 120 μg/mL. Moreover, zedoary oil at 90 and 120 μg/mL increased the ratio of Bax/Bcl-2 protein expression (*P* < 0.01 *vs.* control cells) (Figure 6).

## Discussion

Some zedoary varieties or origins do not contain curdione, curcumol and curzerenone [32-34]. Thus, it is important to determine the compounds in zedoary oil for its quality control [15].

In the present study, the GC-MS results showed that our zedoary oil contained curcumol, β-elemene, curdione and germacrone, which is a characteristic component of zedoary oil [12,35]. Zedoary oil was reported to show antitumor activities toward different human cancer cells and/or animal models with different malignant tumors, such as human oophoroma, hepatocellular carcinoma, lung cancer and leukemia, *in vitro* and *in vivo*[8-11,36]. However, the inhibitory effects of zedoary oil on chronic atrophic gastritis and gastric cancer have not been examined. In our study, inhibitory effects of zedoary oil on the viability of AGS cells and MGC 803 cells were observed. In previous studies [12,32], β-elemene, curcumol and curdione showed inconsistent results for antitumor activities. In the present study, β-elemene, but not curdione and curcumol, showed inhibitory effect on AGS cell proliferation. The ability of β-elemene to inhibit the proliferation of AGS cells was weaker than that of zedoary oil. These findings indicate that other compounds in zedoary oil might have inhibitory effects on AGS cell viability, or that many ingredients may have a synergistic inhibitory effect on AGS cell proliferation, which should be investigated in further studies. Although more than 30 compounds were detected in zedoary oil, we were unable to identify all the compounds and determine the active compounds involved in the inhibition of AGS cell proliferation, owing to time and cost issues.

In this study, zedoary oil had stronger inhibitory effects on AGS cell proliferation in the presence of lower FBS concentrations (0 and 3%). These observations may indicate that some FBS ingredients (*e.g.*, esterases) rapidly break down the active ingredients in zedoary oil, thereby decreasing the inhibitory effects of zedoary oil on the growth of gastric cancer cells. Furthermore, AGS cells may be weaker and more sensitive to chemical compounds because of the lack of nutrition at lower concentrations of FBS [37,38]. We did not investigate the causes of the differences in the inhibitory effects of zedoary oil between the presence and absence of FBS, because cancer cells always exist in a nutrition-rich environment. Zedoary oil showed weaker inhibitory effects on MGC 803 cells than on AGS cells in medium containing 3% FBS, because of differences between the two cell lines.

In our investigations, zedoary oil induced cell cycle arrest at S, G_2_/M and G_0_/G_1_ phases at different times during 6–48 h of treatment. Other compounds, such as tangeretin and nobiletin, have similar effects on the cell cycle [39]. After treatment for 6 and 12 h, cell cycle arrest was induced by zedoary oil at concentrations of 30 and 60 μg/mL, which did not have obvious inhibitory effects on AGS cell proliferation. Cell cycle arrest at 6–12 h may result in DNA repair in AGS cells, with a view to escaping from cell apoptosis/necrosis. Subsequently, after treatment with zedoary oil for 24 h, the cells whose DNA could not be repaired proceeded to cell apoptosis/necrosis, which was confirmed by observations of apoptotic/necrotic characteristics detected by flow cytometry and Hoechst 33342/PI double-staining in zedoary oil-treated AGS cells.

In our experiments, PCNA protein expression in AGS cells was significantly downregulated by zedoary oil treatment. This finding confirmed that zedoary oil inhibited AGS cell proliferation.

Zedoary oil induces cell apoptosis through a mitochondria/caspase-dependent pathway in human hepatoma cells [10]. In the present study, zedoary oil significantly decreased Bcl-2 protein expression and decreased Bax protein expression in 60 μg/mL zedoary oil-treated cells, while the Bax protein level in 120 μg/mL zedoary oil-treated cells was increased, indicating that the balance between Bax and Bcl-2 in cytochrome c-dependent apoptosis was disturbed.

## Conclusions

Zedoary oil inhibited AGS cell proliferation through cell cycle arrest and cell apoptosis promotion, which were related to Bax/Bcl-2 protein expression.

## Abbreviations

DMSO: Dimethyl sulfoxide; PI: Propidium iodine; FBS: Fetal bovine serum; MTT: 3-(4,5-dimethylthizol-2-yl)-2,5-diphenyltetrazolium bromide; TBST: Tris–HCl-buffered saline with 0.1% Tween-20; SDS-PAGE: Sodium dodecyl sulfate-polyacrylamide gel electrophoresis; GC-MS: Gas chromatography–mass spectrometry; GC: Gas chromatography; NIST: National Institute of Standards and Technology; PBS: Phosphate-buffered saline; NC: Nitrocellulose; RT: Retention time; PCNA: Proliferating cell nuclear antigen.

## Competing interests

The authors declare that they have no competing interests.

## Authors’ contributions

GJ and JX designed the study. HS, BT, LL, SS and AC performed the experiments. HS, BT, GJ and JX wrote the manuscript. All authors read and approved the final manuscript.
